# Correlation Between Remote Symptom Reporting by Caregivers and Adverse Clinical Outcomes: Mixed Methods Study

**DOI:** 10.2196/49100

**Published:** 2023-11-21

**Authors:** Ingrid Oakley-Girvan, Reem Yunis, Stephanie J Fonda, Michelle Longmire, Tess L Veuthey, Jennifer Shieh, Sara Aghaee, Ai Kubo, Sharon W Davis, Raymond Liu, Elad Neeman

**Affiliations:** 1 Medable Inc Palo Alto, CA United States; 2 Estenda Solutions Inc Wayne, PA United States; 3 Kaiser Permanente Northern California San Francisco, CA United States; 4 Division of Research Kaiser Permanente Northern California Oakland, CA United States; 5 Department of Hematology Oncology Kaiser Permanente Northern California San Francisco, CA United States; 6 Kaiser Permanente Northern California San Rafael, CA United States

**Keywords:** adverse events, cancer, decentralized clinical trials, electronic patient-reported outcomes, ePROs, mobile health app, observer-reported outcomes, Patient-Reported Outcomes Measurement Information System Patient-Reported Outcome Common Terminology Criteria for Adverse Events, patient-reported outcomes, PRO-CTCAE, PROMIS, remote clinical trials, remote monitoring, smartphone

## Abstract

**Background:**

Timely collection of patient-reported outcomes (PROs) decreases emergency department visits and hospitalizations and increases survival. However, little is known about the outcome predictivity of unpaid informal caregivers’ reporting using similar clinical outcome assessments.

**Objective:**

The aim of this study is to assess whether caregivers and adults with cancer adhered to a planned schedule for electronically collecting patient-reported outcomes (PROs) and if PROs were associated with future clinical events.

**Methods:**

We developed 2 iPhone apps to collect PROs, one for patients with cancer and another for caregivers. We enrolled 52 patient-caregiver dyads from Kaiser Permanente Northern California in a nonrandomized study. Participants used the apps independently for 4 weeks. Specific clinical events were obtained from the patients’ electronic health records up to 6 months following the study. We used logistic and quasi-Poisson regression analyses to test associations between PROs and clinical events.

**Results:**

Participants completed 97% (251/260) of the planned Patient-Reported Outcomes Common Terminology Criteria for Adverse Events (PRO-CTCAE) surveys and 98% (254/260) of the Patient-Reported Outcomes Measurement Information System (PROMIS) surveys. PRO-CTCAE surveys completed by caregivers were associated with patients’ hospitalizations or emergency department visits, grade 3-4 treatment-related adverse events, dose reductions (*P*<.05), and hospice referrals (*P*=.03). PROMIS surveys completed by caregivers were associated with hospice referrals (*P*=.02). PRO-CTCAE surveys completed by patients were not associated with any clinical events, but their baseline PROMIS surveys were associated with mortality (*P*=.03), while their antecedent or final PROMIS surveys were associated with all clinical events examined except for total days of treatment breaks.

**Conclusions:**

In this study, caregivers and patients completed PROs using smartphone apps as requested. The association of caregiver PRO-CTCAE surveys with patient clinical events suggests that this is a feasible approach to reducing patient burden in clinical trial data collection and may help provide early information about increasing symptom severity.

## Introduction

Patient-reported outcomes (PROs) are defined as measures based on a report that comes directly from the patient about the status of their health condition without interpretation by a clinician or anyone else [1]. When used in the oncology setting, PROs can help increase health-related quality of life and decrease emergency department (ED) visits and hospitalizations [2-5]. In some studies, their use was shown to increase survival rates compared with usual care [4,6-8]. The National Cancer Institute developed the PRO version of the Common Terminology Criteria for Adverse Events (PRO-CTCAE) as a tool to provide a standard method to assess symptomatic adverse events (AEs) from the patient’s perspective [9] and funded the Patient-Reported Outcomes Measurement Information System (PROMIS) to measure physical and mental functioning across a variety of diseases to facilitate and improve standardized PRO data collection [10].

Informal caregivers play an integral part in the treatment management of patients with cancer (hereafter referred to as “patients”). Caregivers can observe and report on the patient’s symptoms and daily life. Symptom reporting by caregivers is commonly used in pediatric settings or for patients with cognitive impairments [11,12], but there are few studies of caregiver reporting in adult oncology [13]. Some studies indicate a low-to-moderate correlation between reporting provided by the patient’s caregiver and the patient’s own reported outcomes, with the suggestion that multiple viewpoints may provide additional benefits for patient outcomes [14-16]. Caregivers typically reported more symptoms than clinicians or patients [14,15].

Further highlighting the importance of caregivers’ perspectives, a recent survey of Kaiser Permanente Northern California (KPNC) oncologists (n=38) found that oncologists routinely rely on information from caregivers to predict AEs and symptoms, and when there is a discrepancy between a caregiver and a patient’s report, they rely more heavily on the caregiver’s report [17]. Unfortunately, caregiver reporting is largely informal and collected ad hoc by individual providers. To fill this gap, we created mobile apps and conducted a study to formally assess the power of caregiver reporting.

## Methods

### Ethical Considerations

The KPNC Institutional Review Board (IRB) approved this study (KPNC IRB #1576055). We obtained written informed consent within the app from participants before any data collection. The app was housed on the patients’ iPhones, which were designed by Apple to be password-protected. In addition, the app itself was password-protected and could be opened by either a passcode, facial recognition, or fingerprint, depending on how the user preferred to set it up for controlled access. These biometrics were not available to KPNC or Medable but were stored on the participants’ own phones as part of the Apple iPhone operating system.

The Medable platform use was accessed by Kaiser information technology and technology teams; security and privacy for all data captured by the iPhone and the study as a whole were reviewed and approved by the KPNC IRB. All data collected using the app was stored in a secure HIPAA (Health Insurance Portability and Accountability Act)-compliant cloud that was accessed and controlled by KPNC project staff. The data were deidentified for analysis.

Participants who completed the surveys in their app and 2 semi-structured video conferencing interviews each received a US $100 Amazon gift certificate in appreciation for the time and effort they spent.

### Setting

This study was conducted in collaboration with KPNC, which is an integrated health care delivery system serving over 4.5 million members across the geography of Northern California.

### Study Design

This was a single-arm, longitudinal, mixed methods, prospective study evaluating the feasibility of using mobile health apps for symptom reporting by patients and their informal caregivers. To participate, both the patient and their caregiver had to be eligible. Eligible patients were adult (aged 18 years or older) KPNC members receiving intravenous chemotherapy or immunotherapy for cancer; living with a caregiver who was willing and eligible to participate; speaking English; and owning an iPhone 6 or above. The stage of cancer was not an inclusion or exclusion criterion. Patients with serious mental health concerns or insufficient cognition to consent, as determined by their physician per KPNC policy, were not eligible to participate. Caregivers were identified by eligible patients based on who lived with them and spent the most time providing unpaid care. Eligible caregivers lived with the patient, were aged 18 or older, spoke English, and owned an iPhone 6 or above. There was no requirement for the length of time the caregiver lived with the patient. Patients and caregivers who owned an Android phone were ineligible because, at the time of the study, the apps were available only on the iPhone. Caregivers were not required to be KPNC members.

### Recruitment and Informed Consent

Recruitment and informed consent were completed remotely using email and televisits, along with an electronic in-app signature from both members of the dyad, from October 2020 through March 2021. Recruitment emails were sent to 2155 potential patients. Of the 247 respondents, 166 were determined to be ineligible, 20 declined to participate before eligibility could be confirmed, and 7 declined to participate after learning more details about the study.

A total of 54 patient-caregiver dyads participated in the study; however, 2 dyads were excluded from the analysis because, despite scheduled treatment at enrollment, the patient did not receive any intravenous chemotherapy or immunotherapy treatments during the study period, thus making them ineligible. Therefore, 52 patient-caregiver dyads were included in this analysis.

### Protocol

The 2 smartphone apps, DigiBioMarC and TOGETHERCare, have been described elsewhere [18,19].

Patients and caregivers were asked to complete the PRO-CTCAE and PROMIS surveys at baseline and then once per week, for a total of 5 planned instances of the surveys. From the comfort of their own homes and without medical intervention, caregivers and patients used their iPhones to report on their symptoms. No attempt was made to coordinate the timing of the surveys of caregivers and patients. The apps were designed to provide access to each subsequent survey at set intervals (a minimum of 7 days) dependent upon the completion of the previous survey. The data provided by the participants were neither given to their clinical team nor were the participants given any feedback from clinical staff regarding the survey responses in this study.

### Measures

#### Reporting of Patient Symptoms

In order to test standardized caregiver and patient reporting of patient symptoms, we developed 2 mobile apps: DigiBioMarC for patients and TOGETHERCare for their informal caregivers. These apps enable participation in decentralized clinical trials and remote cancer care by collecting informed consent, electronic patient-reported outcomes (ePROs), surveys, and other digital data [20]. We tested the usability of both apps for approximately 4 weeks with 52 patient-caregiver dyads recruited through KPNC. This analysis assesses whether patients and their informal caregivers completed the apps’ symptom reporting surveys as planned by the study (ie, adherence to the study protocol) and whether the collected data were associated with specific clinical events experienced by some of the patient participants.

#### Clinical Events

Using KPNC electronic health records (EHRs), patients’ clinical events were retrospectively collected from the study period and up to 2 months after. Clinical events included the total number of cancer-related ED visits and hospitalizations (combined into a single measure), the total number of grade 3-4 treatment-related AEs and treatment-dose reductions (combined into a single measure given that dose reductions are often the consequence of such high-grade AEs), and the total number of treatment break days for treatment breaks that lasted 3 or more days. Additionally, mortality (“yes” or “no”) and hospice referrals (“yes” or “no”) were followed for up to 6 months after completion of study participation.

#### Symptom Reporting

A total of 2 questionnaires were chosen for reporting symptoms: 12 symptoms from the PRO-CTCAE and the PROMIS Physical Function Short Form 4a. These questionnaires were used by patients and, with slight wording changes, also by caregivers reporting about their patients. The survey items used by both patients and caregivers are included in Multimedia Appendix 1.

The studied PRO-CTCAE symptoms were chosen by the study team of KPNC oncologists (RL and EN) based on the known or anticipated effects of the therapies in the study patient population. The following symptoms were selected for inclusion in the apps: nausea, anxiety, pain, sadness, vomiting, appetite, constipation, diarrhea, shortness of breath, numbness, insomnia, and fatigue [18]. We created a total count of each severe or very severe symptom reported by the participant on the PRO-CTCAE. Patient-reported symptoms rated as severe and very severe were classified together as severe due to the low numbers of both responses. In consultation with the KPNC IRB and following guidance from the National Cancer Institute [21], we allowed participants to decline to answer questions (for all surveys included).

The PROMIS Physical Function Short Form 4a covered the patient’s physical difficulty (from “unable to do” to “no difficulty”) to perform the following: household chores such as vacuuming, using the stairs, walking, and doing errands such as shopping. The study used the T-scores for the PROMIS measure, which normalizes the raw PROMIS score. Population-based, normalized PROMIS scores have a mean of 50 (SD 10) [22,23].

### Analysis

For evaluation of adherence to the study protocol, we counted the total number of PRO-CTCAE and PROMIS surveys completed by both patients and caregivers, as well as the numbers completed within or after the planned 4-week study period.

Additionally, we compared the respective baseline PRO-CTCAE and PROMIS scores for patient-caregiver dyads that completed their surveys within 24 hours of each other. For comparison of PRO-CTCAE scores, we cross-tabulated the patient-caregiver PRO-CTCAE scores and conducted a Fisher’s exact test for an indication of whether the measures were associated. For comparison of the PROMIS scores, we conducted a 2-tailed *t* test assuming unequal variances as well as calculated a correlation coefficient. This part of the analysis examined the baseline surveys only because the patient-caregiver timing for survey completions tended to differ by more than 24 hours after baseline, which prohibited comparisons due to the changing nature of symptoms during active treatment.

For evaluation of the associations between the surveys and patient clinical outcomes, we used logistic and quasi-Poisson regressions. Logistic regressions were used to calculate odds ratios (odds ratios) and 95% CIs in models evaluating hospice referral and death. Quasi-Poisson regressions were used to calculate relative risks and 95% CIs for all other clinical outcomes. All completed surveys with responses other than “decline to answer” were used in the analyses. A total of 2 regressions were estimated per survey. The first regressed each type of AE on the baseline survey, and the second regressed the AE on the closest survey preceding an event (antecedent). If a category of AE did not occur, the final available survey was used. Logged person-days were included in the quasi-Poisson regressions as an offset to adjust for some participants dying within the time frame within which the other clinical events were tracked. The regressions of the antecedent or final surveys adjusted for whether participants completed more than 4 surveys.

## Results

### Participant Characteristics

Study participant demographics and characteristics are provided in Table 1. Most caregivers were men (32/52, 62%), White (33/52, 64%), and had a college degree (28/52, 54%). Most caregivers were the spouse or partner of the patient participant (40/52, 77%). Patients were predominantly White (33/52, 64%) and women (40/52, 77%) with college degrees (38/52, 73%). Most patients (39/52, 75%) had stage 3 or 4 cancer. The greatest proportion of patient participants had breast cancer (17/52, 33%), followed by gastrointestinal and gynecological (13/52, 25% each), thoracic (6/52, 12%), and other cancer types (3/52, 6%).

**Table 1:** Characteristics of the study participants at baseline (N=52 dyads). Percentages might not sum to 100 due to rounding.

Characteristics		Caregivers	Patients
**Age (years)**			
	Mean (SD)	55 (16)	60 (11)
	Median (IQR)	59 (46-67)	62 (53-68)
**Gender, n (%)**			
	Women	20 (39)	40 (77)
	Men	32 (62)	12 (23)
**Ethnicity, n (%)**			
	Hispanic or Latino	4 (8)	5 (10)
	Not Hispanic or Latino	43 (83)	44 (85)
	Did not respond	5 (10)	3 (6)
**Race, n (%)**			
	White	33 (64)	33 (64)
	Black or African American	3 (6)	4 (8)
	Asian, Native Hawaiian, or other Pacific Islander	6 (12)	6 (12)
	Other or multiple races and ethnicities specified	7 (14)	4 (8)
	Did not respond	3 (6)	5 (10)
**Educational attainment, n (%)**			
	Less than ninth grade	1 (2)	1 (2)
	High school or GED^a^	4 (8)	3 (6)
	Some college, no degree	19 (37)	10 (19)
	Associate’s degree	2 (4)	6 (12)
	Bachelor’s degree	16 (31)	16 (31)
	Master’s degree or higher	10 (19)	16 (31)
**Caregiver relationship to patient, n (%)**			
	Spouse or partner	40 (77)	—^b^
	Child or grandchild	6 (12)	—
	Parent	4 (8)	—
	Friend or other relative	2 (4)	—
**Cancer stage at diagnosis, n (%)**			
	1	—	5 (10)
	2	—	8 (15)
	3	—	18 (35)
	4	—	21 (40)
**Cancer type, n (%)**			
	Breast	—	17 (33)
	Gastrointestinal	—	13 (25)
	Gynecological	—	13 (25)
	Thoracic	—	6 (12)
	Other (skin or genitourinary)	—	3 (6)
**Type of cancer treatment, n (%)**			
	Chemotherapy	—	32 (62)
	Immunotherapy	—	10 (19)
	Multiple therapies or other	—	10 (19)
**Baseline PRO-CTCAE^c^ count**			
	Mean (SD)	1 (2)^d^	1 (2)
	Median (IQR)	0 (0-2)^d^	0 (0-2)
**Baseline normalized PROMIS^e^ count**			
	Mean (SD)	47 (8)^f^	46 (8)
	Median (IQR)	46 (41-57)^f^	45 (39-57)

^a^GED: general educational development.

^b^Not applicable.

^c^PRO-CTCAE: Patient-Reported Outcome Common Terminology Criteria for Adverse Events.

^d^n=46.

^e^PROMIS: Patient-Reported Outcomes Measurement Information System.

^f^n=50.

### Survey Compliance

The overall survey completion rate was greater than 96% for both caregivers and patients and for both the PRO-CTCAE (caregivers completed 251/260, 97% surveys, and patients completed 255/260, 98% surveys) and PROMIS (caregivers completed 254/260, 98% surveys, and patients completed 251/260, 97% surveys) in their respective apps (Table 2). Timely completion of these surveys declined beginning at week 3. However, when we extended the time window for completion to allow additional days, the completions for each survey instance exceeded 96%.

Caregivers chose the “decline to answer” option for at least 1 question in 10% (26/251) of the total completed PRO-CTCAE surveys and in 66% (15/254) of the total completed PROMIS surveys. Patients chose this answer for one or more questions in less than 1% (1/255) and 1% (3/251) of the total completed PRO-CTCAE and PROMIS surveys, respectively.

**Table 2:** Study participants’ survey completions: total, baseline, and week 4 survey instances. The time between survey administrations was set at 7 days. The denominator for the calculation of the percentage for each week was 52.

Survey		Total completed^a^ (ever), n (%)	Baseline, n (%)^b^		Week 4, n (%)^b^	
			Within 4 weeks^c^	After 4 weeks^d^	Within 4 weeks^c^	After 4 weeks^d^
**Caregivers**						
	PRO-CTCAE^e^	251 (97)	52 (100)	—^f^	25 (48)	21 (40)
	PROMIS^g^	254 (98)	52 (100)	—	28 (54)	20 (39)
**Patients**						
	PRO-CTCAE	255 (98)	52 (100)	—	29 (56)	21 (40)
	PROMIS	251 (97)	52 (100)	—	26 (50)	22 (42)

^a^The denominator for the calculation of the percentage for “total completed (ever)” is 260 (52 participants within each group × 5 survey instances).

^b^Number of participants who completed the surveys.

^c^Within 4-week study period = consent date + 33 days for a buffer.

^d^After the buffer of 3 days past the planned study period.

^e^PRO-CTCAE: Patient-Reported Outcome Common Terminology Criteria for Adverse Events.

^f^Not applicable.

^g^PROMIS: Patient-Reported Outcomes Measurement Information System.

### Comparison of Patient-Caregiver Survey Scores

A total of 38 (38/52, 73%) dyads completed the baseline PRO-CTCAE surveys within 24 hours of each other. A total of 19 (19/38, 50%) contemporaneous PRO-CTCAE scores were in perfect agreement, and 32 (32/38, 84%) were in perfect agreement or differed by 1 symptom count (*P*<.001, indicating the measures were associated). Where the baseline PRO-CTCAE scores differed by more than 1 count, a total of 5 patients reported more symptoms than their caregivers, and 1 caregiver reported more symptoms than their patient.

A total of 44 (44/52, 85%) dyads completed the baseline PROMIS surveys within 24 hours of each other. The *t* statistic in the comparison of mean PROMIS survey scores was 0.47 (*P*=.47), and the correlation coefficient was 0.44 (*P*=.003).

### Patient Clinical Outcomes as Recorded in the Electronic Health Record

A total of 13 patients (13/52, 25%) had at least 1 cancer-related hospital or ED visit. Of these 13 patients, 4 had a cancer-related hospital stay (durations of 1, 3, 4, and 5 days). A total of 13 patients (13/52, 25%) had at least 1 treatment-related AE or dose reduction. Total days of breaks in treatment ranged from 7 to 42 and affected 18 (18/52, 35%) patients. A total of 8 patients (8/52, 15%) had a hospice referral, and 7 (7/52, 14%) died during the 6-month follow-up period.

### Associations Between Caregiver-Reported PROs and Patient Clinical Outcomes

The caregiver-reported baseline and antecedent or final PRO-CTCAE surveys were associated with total combined hospital and ED visits, total combined AEs, and dose reductions, but not with treatment breaks. Higher counts of severe or very severe symptoms were associated with greater risks of these outcomes (Table 3). The caregiver-reported baseline PRO-CTCAE counts and PROMIS scores were associated with the presence of a hospice referral (Table 4).

**Table 3:** Associations of clinical events with patient-reported outcomes (PROs) completed by caregivers and patients. Each regression model was estimated separately.

Predictors			Total cancer-related hospital and emergency department visits		Total adverse events and dose reductions		Total days of treatment breaks	
			RR^a^ (95% CI)	*P* value	RR (95% CI)	*P* value	RR (95% CI)	*P* value
**Caregivers**								
	**PRO-CTCAE^b^**							
		Baseline	1.5 (1.1-1.8)	.002	1.5 (1.2-1.8)	<.001	1.0 (0.7-1.4)	.85
		Antecedent or final^c^	1.3 (1.2-1.5)	<.001	1.2 (1.0-1.5)	.03	1.1 (0.8-1.3)	.71
	**PROMIS^d^**							
		Baseline	1.0 (0.9-1.1)	>.99	1.0 (0.9-1.0)	.42	1.0 (0.9-1.1)	.84
		Antecedent or final^c^	1.0 (0.9-1.0)	.33	1.0 (0.9-1.0)	.71	1.0 (0.9-1.0)	.99
**Patients**								
	**PRO-CTCAE**							
		Baseline	1.1 (0.8-1.5)	.46	1.2 (0.9-1.4)	.17	1.0 (0.8-1.3)	.74
		Antecedent or final^c^	1.3 (0.8-2.2)	.29	1.5 (1.0-2.2)	.06	0.9 (0.5-1.3)	.55
	**PROMIS**							
		Baseline	0.9 (0.8-1.0)^e^	.04	1.0 (0.9-1.0)	.14	1.0 (1.0-1.1)	.27
		Antecedent or final^c^	0.9 (0.9-0.9)	<.001	0.9 (0.9-1.0)	.04	1.0 (1.0-1.1)	.46

^a^RR: relative risk.

^b^PRO-CTCAE: Patient-Reported Outcome Common Terminology Criteria for Adverse Events.

^c^Antecedent or final refers to the closest survey preceding a clinical event, or the final survey completed if the patient did not experience an event. The regression models using the antecedent or final surveys include dummy variables indicating whether participant completed 4+ surveys.

^d^PROMIS: Patient-Reported Outcomes Measurement Information System.

^e^Due to rounding, this value is at the limit for the upper bound of a CI indicating a significant *P* value.

**Table 4:** Association of hospice referrals and death with patient-reported outcomes (PROs) completed by caregivers and patients. Each regression model was estimated separately.

Predictors			Hospice referral made		Patient died	
			OR^a^ (95% CI)	*P* value	OR (95% CI)	*P* value
**Caregivers**						
	**PRO-CTCAE^b^**					
		Baseline	1.7 (1.1-2.8)	.03	1.6 (1.0-2.4)	.05
		Antecedent or final^c^	1.3 (0.9-1.9)	.14	1.4 (1.0-2.0)	.07
	**PROMIS^d^**					
		Baseline	0.8 (0.7-1.0)	.02	0.9 (0.8-1.0)	.10
		Antecedent or final^c^	1.0 (0.9-1.0)	.25	1.0 (0.9-1.1)	.54
**Patients **						
	**PRO-CTCAE**					
		Baseline	1.3 (0.9-1.9)	.13	1.4 (0.9-2.0)	.11
		Antecedent or final^c^	1.5 (0.8-2.5)	.17	1.4 (0.8-2.5)	.19
	**PROMIS**					
		Baseline	0.9 (0.8-1.0)	.09	0.8 (0.7-1.0)	.03
		Antecedent or final^c^	0.9 (0.8-1.0)	.02	0.7 (0.6-0.9)	.008

^a^OR: odds ratio.

^b^PRO-CTCAE: Patient-Reported Outcome Common Terminology Criteria for Adverse Events.

^c^Antecedent or final refers to the closest survey preceding a clinical event, or the final survey completed if the patient did not experience an event. The regression models using the antecedent or final surveys include dummy variables indicating whether participant completed 4+ surveys.

^d^PROMIS: Patient-Reported Outcomes Measurement Information System.

### Associations Between PROs and Patient Clinical Outcomes

The patients’ self-reported PRO-CTCAE surveys were not associated with any of the clinical events tracked in this study. Conversely, patient self-reported baseline PROMIS surveys were associated with total cancer-related hospital and ED visits and mortality. The antecedent or final patient self-reported PROMIS surveys were associated with all tracked clinical events except total days of treatment breaks (Tables 3 and 4).

## Discussion

### Overview

We present findings of symptom reporting in oncology patients by either the patients themselves or by their informal caregivers using their respective smartphone apps (the DigiBioMarC app for patients and the TOGETHERCare app for informal caregivers). In this study, we demonstrated that patients and caregivers were willing and able to adhere to smartphone app completion of PRO-CTCAE-based symptom reporting and PROMIS surveys over time. We also observed the relationships between these measures and specific subsequent patient clinical events. This study provides valuable insights into the critical nature of caregiver-reported outcomes for early information related to patients’ symptoms and showcases that caregivers can provide high-quality data for remote monitoring of patients’ well-being.

The objective of this study was to obtain 5 instances (per participant) of completed surveys, so we allowed participants to continue in the study beyond the originally planned 4 weeks of participation. When assessing completion against the 4-week time line, survey completion declined over time. However, if we allocated additional time for some participants to complete the surveys, adherence was 97%-98%. Caregivers completed 251 out of 260 PRO-CTCAE surveys, and patients completed 255 out of 260. Caregivers completed 254 out of 260 PROMIS surveys, and patients completed 251 out of 260. The observed decline in timely completion may have been due to the fixed intervals between surveys. This resulted in a time shift forward if a participant did not complete each survey as soon as it was available to them because each survey was triggered by the previous survey’s completion date. This situation extended the study time line by a few days, a week, or more if earlier surveys were not completed in a timely manner. The decline may also have been due to a lack of time-specific reminders, as these were not built into the app, and there was no direct follow-up from research or clinical staff, which was intended to ensure a low clinical team burden.

Despite having the option of “decline to answer” for each survey item, patients overwhelmingly chose to report on their symptoms. While caregivers selected this answer more frequently than patients, some caregivers reported in the semistructured interviews that they chose this option for symptoms they perceived as not applicable or those of which they were unaware.

The caregivers’ assessments of patients’ symptoms using PRO-CTCAE surveys were associated with cancer-related hospital and ED visits, grade 3-4 treatment-related AEs, and treatment-dose reductions. Lower caregiver baseline PROMIS scores regarding the patient’s general physical and mental well-being were associated with referral to hospice. In contrast, the patient’s own PRO-CTCAE surveys were not associated with any of the AEs, although their PROMIS surveys were. This suggests that remote monitoring of symptoms reported by caregivers could be used to prompt increased interaction between the clinical team and the patient, which could in turn reduce poor outcomes and clinical trial dropout.

There was an association between the baseline patient and caregiver PRO-CTCAE surveys, but it was an imperfect one because only 50% (26/52) of dyads agreed completely on the total number of severe or very severe symptoms. The relationship between the baseline PROMIS surveys was even less clear, with a 2-tailed *t* test suggesting no relationship and a correlation coefficient suggesting a moderate relationship. The 2 groups’ differing results for the analysis of the baseline surveys’ relationship to future clinical outcomes may be explained by the discordance in their assessments of the patients’ well-being as reported by the surveys used in the apps. However, the goal of the study was not to determine who is a better reporter of patients’ experiences but to verify that caregiver assessments are informative and valid, with potential for remote patient monitoring.

This fully remote study appears to be unique in assessing whether caregiver-reported symptoms were associated with adult patients’ AEs. The existing literature comparing caregiver PROs with patient PROs examines adult caregivers for pediatric patients [11,12,14,15]. Reeve and colleagues [24] argue for the importance of the caregiver perspective in reporting what an ill child is experiencing; however, we were unable to find studies comparing the correlation between caregiver-reported symptoms and adult patients’ AEs during standard care or in a clinical trial.

This study is also unique in its use of smartphone apps to collect the same symptom and physical and mental well-being data from both patients and caregivers over a similar time frame and for its flexible design. While ePROs have been extensively collected from cancer patients, many of these have been from web-based or tablet use rather than from a smartphone [25-27]. Our smartphone apps were designed to be adaptable to many therapeutic indications. Other smartphone apps do not include PRO-based reporting by caregivers and have been designed for collecting PROs from patients for specific treatment regimens or diagnoses [28-33].

### Limitations

This study has several limitations. First, the sample was composed of 52 dyads, and some study participants did not provide responses to all questions for all surveys. In the design of this single-arm nonintervention study, we focused on having at least 45 dyads complete the study to assess compliance with data collection, and we exceeded that goal. We did not conduct power calculations as we were not focused on an effect size but rather on providing early insight into the utility of remote symptom and health monitoring as predictors of clinical outcomes. We recognize that a randomized controlled study is an essential next step in order to examine the clinical utility of our apps. Third, the study was not intended, designed, or powered to conduct a comparison of the predictive value of patient- versus caregiver-reported outcomes over time. Any differences we observed between the 2 reporters cannot be formally tested. It is possible (but not determined by the project team) that some patient-caregiver dyads collaborated on the completion of their surveys, which would mean the responses were not independent in all cases. Fourth, we used ePRO tools in the TOGETHERCare app for caregivers to report about their patient’s symptoms (PRO-CTCAE) and physical and mental health (PROMIS), and although the results are suggestive that our innovative approach could reduce patient burdens and provide reliable data in remote settings, these tools have not been well tested for use by caregivers. Fifth, most patients in the study were women with high education and literacy levels, and only participants who spoke English and owned an iPhone were eligible to participate. Android versions of the apps have been developed since this study was completed, and we plan to continue the testing efforts to expand population representation.

### Strengths

This study has several strengths. First, observations were collected from both patients and their caregivers while patients were in active treatment for cancer requiring intravenous chemotherapy or immunotherapy, both of which are known to result in frequent AEs and cause symptoms. Thus, we believe that the study has good face validity and clinical relevance. Second, we used KPNC’s EHR data on hospital and ED visits, AEs and dose reductions, total days of treatment breaks, hospice referrals, and deaths. Because KPNC is an integrated health care delivery system in which patients receive virtually all their care, we have confidence that this accurately captures all patient-related events with fidelity across the participating patient population. Third, because participants used their own iPhones, there was less of a learning curve than there might have been with a provisioned device. “Bring Your Own Device” has been shown to be advantageous in terms of data collection [34], and this work would need to be validated in a population with provisioned devices should those be important for specific clinical trials.

### Conclusions

In this study, caregivers and patients completed PROs using smartphone apps as requested. Caregivers and patients reporting of patients’ health status was associated with patients’ clinical events. In particular, the association of caregiver-reported PRO-CTCAE surveys with patient clinical events suggests that this is a feasible approach to reducing patient burden in clinical trial data collection and may help provide early information about increasing symptom severity.

Because caregiver reporting of patients’ symptoms and physical function provided insights ahead of adverse clinical events, it could be advantageous for clinical trials and clinical care teams to incorporate caregiver observations. Incorporating caregiver observations may reduce the burden on patients and improve clinical insight into patients’ experiences outside the clinical setting. This is especially important for high-acuity diseases that require a significant level of care management. It is worth considering the inclusion of informal caregivers to help report on patient symptoms and function in adult clinical trials, particularly ones conducted partially or completely remotely. This could help reduce the burden on patients, facilitate earlier clinical action for symptoms with increasing intensity, and ultimately reduce trial dropout.

## Appendix

Multimedia Appendix 1 Survey items used by patients and caregivers.

## Abbreviations

AE: adverse event
ED: emergency department
EHR: electronic health record
ePRO: electronic patient-reported outcome
HIPAA: Health Insurance Portability and Accountability Act
IRB: Institutional Review Board
KPNC: Kaiser Permanente Northern California
mHealth: mobile health
PRO: patient-reported outcome
PRO-CTCAE: Patient-Reported Outcome Common Terminology Criteria for Adverse Events
PROMIS: Patient-Reported Outcomes Measurement Information System

## Data Availability

The data sets generated during or analyzed during this study are not publicly available due to the sensitive nature of the data captured, the data release requirements of the KPNC subcontract, and the intellectual property associated with this work, but are available from the corresponding author on reasonable request.
