# Correction: Correlation Between Remote Symptom Reporting by Caregivers and Adverse Clinical Outcomes: Mixed Methods Study

**DOI:** 10.2196/56368

**Published:** 2024-01-30

**Authors:** Ingrid Oakley-Girvan, Reem Yunis, Stephanie J Fonda, Michelle Longmire, Tess L Veuthey, Jennifer Shieh, Sara Aghaee, Ai Kubo, Sharon W Davis, Raymond Liu, Elad Neeman

**Affiliations:** 1 Medable Inc Palo Alto, CA United States; 2 Estenda Solutions Inc Wayne, PA United States; 3 Kaiser Permanente Northern California San Francisco, CA United States; 4 Division of Research Kaiser Permanente Northern California Oakland, CA United States; 5 Department of Hematology Oncology Kaiser Permanente Northern California San Francisco, CA United States; 6 Kaiser Permanente Northern California San Rafael, CA United States

In “Correlation Between Remote Symptom Reporting by Caregivers and Adverse Clinical Outcomes: Mixed Methods Study” (J Med Internet Res. 2023 Nov 21:25:e49100) the authors Sara Aghaee, Ai Kubo, and Raymond Liu were listed with the incorrect affiliations. The author Raymond Liu was also missing an affiliation.

In the original manuscript, the affiliations for the authors appeared as follows:

Sara Aghaee: Department of Hematology Oncology, The Kaiser Permanente Medical Group, San Francisco, CA, United States

Ai Kubo: Department of Hematology Oncology, The Kaiser Permanente Medical Group, San Francisco, CA, United States

Raymond Liu: Department of Hematology Oncology, The Kaiser Permanente Medical Group, San Francisco, CA, United States

This has been corrected to:

Sara Aghaee: Division of Research, Kaiser Permanente Northern California, Oakland, CA, USA

Ai Kubo: Division of Research, Kaiser Permanente Northern California, Oakland, CA, USA

Raymond Liu: Department of Hematology Oncology, Kaiser Permanente Northern California, San Francisco, CA, United States

Additionally, the following affiliation has been included for the author Raymond Liu:

Division of Research, Kaiser Permanente Northern California, Oakland, CA, United States

The correction will appear in the online version of the paper on the JMIR Publications website on January 30, 2024, together with the publication of this correction notice. Because this was made after submission to PubMed, PubMed Central, and other full-text repositories, the corrected article has also been resubmitted to those repositories.

