# Supplementary material for: Correlation Between Remote Symptom Reporting by Caregivers and Adverse Clinical Outcomes: Mixed Methods Study
Source: J Med Internet Res. 2023 Nov 21;25:e49100. doi: 10.2196/49100 (PMC10698661; doi:10.2196/49100)
Supplement: Multimedia Appendix 1 [file jmir_v25i1e49100_app1.docx]

**Supplemental Materials**

**Survey items for caregivers about their patient**

**Loved One’s Short Symptom Report** *[PRO_CTCAE—Baseline (BL), Days 7, 14, 21, & 28]*

**As individuals go through treatment for their cancer they sometimes experience different symptoms and side effects. For each question, please select the one response that you feel best describes your loved one’s experiences over the past 7 days…**

1. **In the last 7 days, what was the SEVERITY of your loved one’s DECREASED APPETITE at its** **WORST?**

| ○ None | ○ Mild | ○ Moderate | ○ Severe | ○ Very severe | ○ Decline to answer |
| --- | --- | --- | --- | --- | --- |

- 1. ***[IF >NONE*] In the last 7 days, how much did DECREASED APPETITE INTERFERE with your loved one’s usual or daily activities?**

| ○ Not at all | ○ A little bit | ○ Somewhat | ○ Quite a bit | ○ Very much | ○ Decline to answer |
| --- | --- | --- | --- | --- | --- |

1. **In the last 7 days, how OFTEN did your loved one have NAUSEA?**

| ○ Never | ○ Rarely | ○ Occasionally | ○ Frequently | ○ Almost constantly | ○ Decline to answer |
| --- | --- | --- | --- | --- | --- |

| ○ None | ○ Mild | ○ Moderate | ○ Severe | ○ Very severe | ○ Decline to answer |
| --- | --- | --- | --- | --- | --- |

1. ***[IF >NEVER]* In the last 7 days, what was the SEVERITY of your loved one’s NAUSEA at its WORST?**
2. **In the last 7 days, how OFTEN did your loved one have VOMITING?**

| \| ○ Never \| ○ Rarely \| ○ Occasionally \| ○ Frequently \| ○ Almost constantly \| ○ Decline to answer \| \| --- \| --- \| --- \| --- \| --- \| --- \| |  |  |  |  |  |  |
| --- | --- | --- | --- | --- | --- | --- | --- | --- | --- | --- | --- | --- |

1. ***[IF > NEVER]* In the last 7 days, what was the SEVERITY of your loved one’s VOMITING at its WORST?**

| ○ None | ○ Mild | ○ Moderate | ○ Severe | ○ Very severe | ○ Decline to answer |
| --- | --- | --- | --- | --- | --- |

1. **In the last 7 days, what was the SEVERITY of your loved one’s CONSTIPATION at its WORST?**

| \| ○ None \| ○ Mild \| ○ Moderate \| ○ Severe \| ○ Very severe \| ○ Decline to answer \| \| --- \| --- \| --- \| --- \| --- \| --- \| |  |  |  |  |  |
| --- | --- | --- | --- | --- | --- | --- | --- | --- | --- | --- | --- |

1. **In the last 7 days, how OFTEN did your loved one have LOOSE OR WATERY STOOLS (DIARRHEA)?**

| ○ Never | ○ Rarely | ○ Occasionally | ○ Frequently | ○ Almost constantly | ○ Decline to answer |
| --- | --- | --- | --- | --- | --- |

1. **In the last 7 days, what was the SEVERITY of your loved one’s SHORTNESS OF BREATH at its WORST?**

| ○ None | ○ Mild | ○ Moderate | ○ Severe | ○ Very severe | ○ Decline to answer |
| --- | --- | --- | --- | --- | --- |

| ○ Not at all | ○ A little bit | ○ Somewhat | ○ Quite a bit | ○ Very much | ○ Decline to answer |
| --- | --- | --- | --- | --- | --- |

1. ***[IF >NONE]* In the last 7 days, how much did SHORTNESS OF BREATH INTERFERE with your loved one’s usual or daily activities?**
2. **In the last 7 days, what was the SEVERITY of your loved one’s NUMBNESS OR TINGLING IN THEIR HANDS OR FEET at its WORST?**

| ○ None | ○ Mild | ○ Moderate | ○ Severe | ○ Very severe | ○ Decline to answer |
| --- | --- | --- | --- | --- | --- |

1. ***[IF >NONE]* In the last 7 days, how much did NUMBNESS OR TINGLING IN THEIR HANDS OR FEET INTERFERE with your loved one’s usual or daily activities?**

| ○ Not at all | ○ A little bit | ○ Somewhat | ○ Quite a bit | ○ Very much | ○ Decline to answer |
| --- | --- | --- | --- | --- | --- |

1. **In the last 7 days, how OFTEN did your loved one have PAIN?**

| ○ Never | ○ Rarely | ○ Occasionally | ○ Frequently | ○ Almost constantly | ○ Decline to answer |
| --- | --- | --- | --- | --- | --- |

1. ***[IF >NEVER]* In the last 7 days, what was the SEVERITY of your loved one’s PAIN at its WORST?**

| ○ None | ○ Mild | ○ Moderate | ○ Severe | ○ Very severe | ○ Decline to answer |
| --- | --- | --- | --- | --- | --- |

| ○ Not at all | ○ A little bit | ○ Somewhat | ○ Quite a bit | ○ Very much | ○ Decline to answer |
| --- | --- | --- | --- | --- | --- |

1. ***[IF >NONE]* In the last 7 days, how much did PAIN INTERFERE with your loved one’s usual or daily activities?**
2. **In the last 7 days, what was the SEVERITY of your loved one’s INSOMNIA (INCLUDING DIFFICULTY FALLING ASLEEP, STAYING ASLEEP, OR WAKING UP EARLY) at its WORST?**

| ○ None | ○ Mild | ○ Moderate | ○ Severe | ○ Very severe | ○ Decline to answer |
| --- | --- | --- | --- | --- | --- |

1. ***[IF >NONE]* In the last 7 days, how much did INSOMNIA (INCLUDING DIFFICULTY FALLING ASLEEP, STAYING ASLEEP, OR WAKING UP EARLY) INTERFERE with your loved one’s usual or daily activities?**

| ○ Not at all | ○ A little bit | ○ Somewhat | ○ Quite a bit | ○ Very much | ○ Decline to answer |
| --- | --- | --- | --- | --- | --- |

1. **In the last 7 days, what was the SEVERITY of your loved one’s FATIGUE, TIREDNESS, OR LACK OF ENERGY at its WORST?**

| ○ None | ○ Mild | ○ Moderate | ○ Severe | ○ Very severe | ○ Decline to answer |
| --- | --- | --- | --- | --- | --- |

1. ***[IF >NONE]* In the last 7 days, how much did FATIGUE, TIREDNESS, OR LACK OF ENERGY INTERFERE with your loved one’s usual or daily activities?**

| ○ Not at all | ○ A little bit | ○ Somewhat | ○ Quite a bit | ○ Very much | ○ Decline to answer |
| --- | --- | --- | --- | --- | --- |

1. **In the last 7 days, how OFTEN did your loved one feel ANXIETY?**

| ○ Never | ○ Rarely | ○ Occasionally | ○ Frequently | ○ Almost constantly | ○ Decline to answer |
| --- | --- | --- | --- | --- | --- |

1. ***[IF >NEVER*] In the last 7 days, what was the SEVERITY of your loved one’s ANXIETY at its WORST?**

| ○ None | ○ Mild | ○ Moderate | ○ Severe | ○ Very severe | ○ Decline to answer |
| --- | --- | --- | --- | --- | --- |

1. ***[IF >NONE]* In the last 7 days, how much did ANXIETY INTERFERE with your loved one’s usual or daily activities?**

| ○ Not at all | ○ A little bit | ○ Somewhat | ○ Quite a bit | ○ Very much | ○ Decline to answer |
| --- | --- | --- | --- | --- | --- |

1. **In the last 7 days, how OFTEN did your loved one have SAD OR UNHAPPY FEELINGS?**

| ○ Never | ○ Rarely | ○ Occasionally | ○ Frequently | ○ Almost constantly | ○ Decline to answer |
| --- | --- | --- | --- | --- | --- |

1. ***[IF >NEVER]* In the last 7 days, what was the SEVERITY of your loved one’s SAD OR UNHAPPY FEELINGS at its WORST?**

| ○ None | ○ Mild | ○ Moderate | ○ Severe | ○ Very severe | ○ Decline to answer |
| --- | --- | --- | --- | --- | --- |

1. ***[IF >NONE*] In the last 7 days, how much did SAD OR UNHAPPY FEELINGS INTERFERE with your loved one’s usual or daily activities?**

| ○ Not at all | ○ A little bit | ○ Somewhat | ○ Quite a bit | ○ Very much | ○ Decline to answer |
| --- | --- | --- | --- | --- | --- |

1. **Are there any other symptoms that your loved one has that you wish to report?**

| ○ Yes | ○ No | ○ Decline to answer |
| --- | --- | --- |

1. ***[IF YES ABOVE]* Please list any other symptoms:**

|  | **In the last 7 days, what was the SEVERITY of this symptom at its WORST?** | | | | | |
| --- | --- | --- | --- | --- | --- | --- |
|  | ○ None | ○ Mild | ○ Moderate | ○ Severe | ○ Very severe | ○ Decline to answer |
|  | ○ None | ○ Mild | ○ Moderate | ○ Severe | ○ Very severe | ○ Decline to answer |
|  | ○ None | ○ Mild | ○ Moderate | ○ Severe | ○ Very severe | ○ Decline to answer |
|  | ○ None | ○ Mild | ○ Moderate | ○ Severe | ○ Very severe | ○ Decline to answer |
|  | ○ None | ○ Mild | ○ Moderate | ○ Severe | ○ Very severe | ○ Decline to answer |

**Fast 4** *[PROMIS Item Bank v2.0 – Physical Function – Short Form 4a—BL, Days 7, 14, 21, & 28]*

1. **Please respond to each question or statement by selecting one response per row. *Is your loved one able to:***

| 1. **do chores such as vacuuming or yard work?** | ○ Without any difficulty | ○ With a little difficulty | ○ With some difficulty | ○ With much difficulty | ○ Unable to do | ○ Decline to answer |
| --- | --- | --- | --- | --- | --- | --- |
| 1. **go up and down stairs at a normal pace?** | ○ Without any difficulty | ○ With a little difficulty | ○ With some difficulty | ○ With much difficulty | ○ Unable to do | ○ Decline to answer |
| 1. **go for a walk of at least 15 minutes?** | ○ Without any difficulty | ○ With a little difficulty | ○ With some difficulty | ○ With much difficulty | ○ Unable to do | ○ Decline to answer |
| 1. **run errands and shop?** | ○ Without any difficulty | ○ With a little difficulty | ○ With some difficulty | ○ With much difficulty | ○ Unable to do | ○ Decline to answer |

**Survey items for patients**

**Short symptom report** *[PRO_CTCAE—Baseline (BL), Days 7, 14, 21, & 28]*

**As individuals go through treatment for their cancer they sometimes experience different symptoms and side effects. For each question, please select the one response that best describes your experiences over the past 7 days…**

1. **In the last 7 days, what was the SEVERITY of your DECREASED APPETITE at its** **WORST?**

| ○ None | ○ Mild | ○ Moderate | ○ Severe | ○ Very severe | ○ Decline to answer |
| --- | --- | --- | --- | --- | --- |

1. ***[IF >NONE*] In the last 7 days, how much did DECREASED APPETITE INTERFERE with your usual or daily activities?**

| ○ Not at all | ○ A little bit | ○ Somewhat | ○ Quite a bit | ○ Very much | ○ Decline to answer |
| --- | --- | --- | --- | --- | --- |

1. **In the last 7 days, how OFTEN did you have NAUSEA?**

| ○ Never | ○ Rarely | ○ Occasionally | ○ Frequently | ○ Almost constantly | ○ Decline to answer |
| --- | --- | --- | --- | --- | --- |

1. ***[IF >NEVER*] In the last 7 days, what was the SEVERITY of your NAUSEA at its WORST?**

| ○ None | ○ Mild | ○ Moderate | ○ Severe | ○ Very severe | ○ Decline to answer |
| --- | --- | --- | --- | --- | --- |

1. **In the last 7 days, how OFTEN did you have VOMITING?**

| \| ○ Never \| ○ Rarely \| ○ Occasionally \| ○ Frequently \| ○ Almost constantly \| ○ Decline to answer \| \| --- \| --- \| --- \| --- \| --- \| --- \| |  |  |  |  |  |  |
| --- | --- | --- | --- | --- | --- | --- | --- | --- | --- | --- | --- | --- |

1. ***[IF >NEVER*] In the last 7 days, what was the SEVERITY of your VOMITING at its WORST?**

| ○ None | ○ Mild | ○ Moderate | ○ Severe | ○ Very severe | ○ Decline to answer |
| --- | --- | --- | --- | --- | --- |

1. **In the last 7 days, what was the SEVERITY of your CONSTIPATION at its WORST?**

| \| ○ None \| ○ Mild \| ○ Moderate \| ○ Severe \| ○ Very severe \| ○ Decline to answer \| \| --- \| --- \| --- \| --- \| --- \| --- \| |  |  |  |  |  |
| --- | --- | --- | --- | --- | --- | --- | --- | --- | --- | --- | --- |

1. **In the last 7 days, how OFTEN did you have LOOSE OR WATERY STOOLS (DIARRHEA)?**

| ○ Never | ○ Rarely | ○ Occasionally | ○ Frequently | ○ Almost constantly | ○ Decline to answer |
| --- | --- | --- | --- | --- | --- |

1. **In the last 7 days, what was the SEVERITY of your SHORTNESS OF BREATH at its WORST?**

| ○ None | ○ Mild | ○ Moderate | ○ Severe | ○ Very severe | ○ Decline to answer |
| --- | --- | --- | --- | --- | --- |

1. ***[IF >NONE*] In the last 7 days, how much did SHORTNESS OF BREATH INTERFERE with your usual or daily activities?**

| ○ Not at all | ○ A little bit | ○ Somewhat | ○ Quite a bit | ○ Very much | ○ Decline to answer |
| --- | --- | --- | --- | --- | --- |

1. **In the last 7 days, what was the SEVERITY of your NUMBNESS OR TINGLING IN YOUR HANDS OR FEET at its WORST?**

| ○ None | ○ Mild | ○ Moderate | ○ Severe | ○ Very severe | ○ Decline to answer |
| --- | --- | --- | --- | --- | --- |

1. ***[IF >NONE*] In the last 7 days, how much did NUMBNESS OR TINGLING IN YOUR HANDS OR FEET INTERFERE with your usual or daily activities?**

| ○ Not at all | ○ A little bit | ○ Somewhat | ○ Quite a bit | ○ Very much | ○ Decline to answer |
| --- | --- | --- | --- | --- | --- |

1. **In the last 7 days, how OFTEN did you have PAIN?**

| ○ Never | ○ Rarely | ○ Occasionally | ○ Frequently | ○ Almost constantly | ○ Decline to answer |
| --- | --- | --- | --- | --- | --- |

1. ***[IF >NEVER*] In the last 7 days, what was the SEVERITY of your PAIN at its WORST?**

| ○ None | ○ Mild | ○ Moderate | ○ Severe | ○ Very severe | ○ Decline to answer |
| --- | --- | --- | --- | --- | --- |

1. ***[IF >NONE*] In the last 7 days, how much did PAIN INTERFERE with your usual or daily activities?**

| ○ Not at all | ○ A little bit | ○ Somewhat | ○ Quite a bit | ○ Very much | ○ Decline to answer |
| --- | --- | --- | --- | --- | --- |

1. **In the last 7 days, what was the SEVERITY of your INSOMNIA (INCLUDING DIFFICULTY FALLING ASLEEP, STAYING ASLEEP, OR WAKING UP EARLY) at its WORST?**

| ○ None | ○ Mild | ○ Moderate | ○ Severe | ○ Very severe | ○ Decline to answer |
| --- | --- | --- | --- | --- | --- |

1. ***[IF >NONE*] In the last 7 days, how much did INSOMNIA (INCLUDING DIFFICULTY FALLING ASLEEP, STAYING ASLEEP, OR WAKING UP EARLY) INTERFERE with your usual or daily activities?**

| ○ Not at all | ○ A little bit | ○ Somewhat | ○ Quite a bit | ○ Very much | ○ Decline to answer |
| --- | --- | --- | --- | --- | --- |

1. **In the last 7 days, what was the SEVERITY of your FATIGUE, TIREDNESS, OR LACK OF ENERGY at its WORST?**

| ○ None | ○ Mild | ○ Moderate | ○ Severe | ○ Very severe | ○ Decline to answer |
| --- | --- | --- | --- | --- | --- |

1. ***[IF >NONE*] In the last 7 days, how much did FATIGUE, TIREDNESS, OR LACK OF ENERGY INTERFERE with your usual or daily activities?**

| ○ Not at all | ○ A little bit | ○ Somewhat | ○ Quite a bit | ○ Very much | ○ Decline to answer |
| --- | --- | --- | --- | --- | --- |

1. **In the last 7 days, how OFTEN did you feel ANXIETY?**

| ○ Never | ○ Rarely | ○ Occasionally | ○ Frequently | ○ Almost constantly | ○ Decline to answer |
| --- | --- | --- | --- | --- | --- |

1. ***[IF >*** ***NEVER*] In the last 7 days, what was the SEVERITY of your ANXIETY at its WORST?**

| ○ None | ○ Mild | ○ Moderate | ○ Severe | ○ Very severe | ○ Decline to answer |
| --- | --- | --- | --- | --- | --- |

1. ***[IF >NONE*] In the last 7 days, how much did ANXIETY INTERFERE with your usual or daily activities?**

| ○ Not at all | ○ A little bit | ○ Somewhat | ○ Quite a bit | ○ Very much | ○ Decline to answer |
| --- | --- | --- | --- | --- | --- |

1. **In the last 7 days, how OFTEN did you have SAD OR UNHAPPY FEELINGS?**

| ○ Never | ○ Rarely | ○ Occasionally | ○ Frequently | ○ Almost constantly | ○ Decline to answer |
| --- | --- | --- | --- | --- | --- |

1. ***[IF >*** ***NEVER*] In the last 7 days, what was the SEVERITY of your SAD OR UNHAPPY FEELINGS at its WORST?**

| ○ None | ○ Mild | ○ Moderate | ○ Severe | ○ Very severe | ○ Decline to answer |
| --- | --- | --- | --- | --- | --- |

1. ***[IF >NONE*] In the last 7 days, how much did SAD OR UNHAPPY FEELINGS INTERFERE with your usual or daily activities?**

| ○ Not at all | ○ A little bit | ○ Somewhat | ○ Quite a bit | ○ Very much | ○ Decline to answer |
| --- | --- | --- | --- | --- | --- |

1. **Do you have any other symptoms that you wish to report?**

| ○ Yes | ○ No | ○ Decline to answer |
| --- | --- | --- |

1. ***[IF YES ABOVE]* Please list any other symptoms:**

|  | In the last 7 days, what was the SEVERITY of this symptom at its WORST? | | | | | | |
| --- | --- | --- | --- | --- | --- | --- | --- |
|  | ○ None | ○ Mild | ○ Moderate | ○ Severe | ○ Very severe | ○ Decline to answer |  |
|  | ○ None | ○ Mild | ○ Moderate | ○ Severe | ○ Very severe | ○ Decline to answer |  |
|  | ○ None | ○ Mild | ○ Moderate | ○ Severe | ○ Very severe | ○ Decline to answer |  |
|  | ○ None | ○ Mild | ○ Moderate | ○ Severe | ○ Very severe | ○ Decline to answer |  |
|  | ○ None | ○ Mild | ○ Moderate | ○ Severe | ○ Very severe | ○ Decline to answer |  |

**Fast 4** *[PROMIS Item Bank v2.0 – Physical Function – Short Form 4a—BL, Days 7, 14, 21, & 28]*

1. **Please respond to each question or statement by selecting one response per row.**

| 1. **Are you able to do chores such as vacuuming or yard work?** | ○ Without any difficulty | ○ With a little difficulty | ○ With some difficulty | ○ With much difficulty | ○ Unable to do | ○ Decline to answer |
| --- | --- | --- | --- | --- | --- | --- |
| 1. **Are you able to go up and down stairs at a normal pace?** | ○ Without any difficulty | ○ With a little difficulty | ○ With some difficulty | ○ With much difficulty | ○ Unable to do | ○ Decline to answer |
| 1. **Are you able to go for a walk of at least 15 minutes?** | ○ Without any difficulty | ○ With a little difficulty | ○ With some difficulty | ○ With much difficulty | ○ Unable to do | ○ Decline to answer |
| 1. **Are you able to run errands and shop?** | ○ Without any difficulty | ○ With a little difficulty | ○ With some difficulty | ○ With much difficulty | ○ Unable to do | ○ Decline to answer |
